# Simplified and Rapid Determination of Primaquine and 5,6-Orthoquinone Primaquine by UHPLC-MS/MS: Its Application to a Pharmacokinetic Study

**DOI:** 10.3390/molecules26144357

**Published:** 2021-07-19

**Authors:** Waritda Pookmanee, Siriwan Thongthip, Jeeranut Tankanitlert, Mathirut Mungthin, Chonlaphat Sukasem, Supeecha Wittayalertpanya

**Affiliations:** 1 Interdisciplinary Program in Pharmacology, Graduate School, Chulalongkorn University, Bangkok 10330, Thailand; waritda@hotmail.com; 2 Clinical Pharmacokinetics and Pharmacogenomics Research Unit, Faculty of Medicine, Chulalongkorn University, Bangkok 10330, Thailand; 3 Maha Chakri Sirindhorn Clinical Research Center Under the Royal Patronage, Faculty of Medicine, Chulalongkorn University, Bangkok 10330, Thailand; siriwan.j@chulacrc.org; 4 Department of Pharmacology, Phramongkutklao College of Medicine, Bangkok 10400, Thailand; jeeranut.tan@pcm.ac.th (J.T.); mathirut@hotmail.com (M.M.); 5 Division of Pharmacogenomics and Personalized Medicine, Department of Pathology, Faculty of Medicine Ramathibodi Hospital, Mahidol University, Bangkok 10400, Thailand; chonlaphat.suk@mahidol.ac.th; 6 Laboratory for Pharmacogenomics, Somdech Phra Debaratana Medical Center (SDMC), Ramathibodi Hospital, Bangkok 10400, Thailand; 7 Pharmacogenomics and Precision Medicine, The Preventive Genomics & Family Check-up Services Center, Bumrungrad International Hospital, Bangkok 10110, Thailand; 8 Department of Pharmacology, Faculty of Medicine, Chulalongkorn University, Bangkok 10330, Thailand

**Keywords:** primaquine, 5,6-orthoquinone-primaquine, plasma, urine

## Abstract

The method for the determination of primaquine (PQ) and 5,6-orthoquinone primaquine (5,6-PQ), the representative marker for PQ active metabolites, via CYP2D6 in human plasma and urine has been validated. All samples were extracted using acetonitrile for protein precipitation and analyzed using the ultra-high-performance liquid chromatography–tandem mass spectrometry (UHPLC-MS/MS) system. Chromatography separation was carried out using a Hypersil GOLD^TM^ aQ C_18_ column (100 × 2.1 mm, particle size 1.9 μm) with a C_18_ guard column (4 × 3 mm) flowed with an isocratic mode of methanol, water, and acetonitrile in an optimal ratio at 0.4 mL/min. The retention times of 5,6-PQ and PQ in plasma and urine were 0.8 and 1.6 min, respectively. The method was validated according to the guideline. The linearity of the analytes was in the range of 25–1500 ng/mL. The matrix effect of PQ and 5,6-PQ ranged from 100% to 116% and from 87% to 104% for plasma, and from 87% to 89% and from 86% to 87% for urine, respectively. The recovery of PQ and 5,6-PQ ranged from 78% to 95% and form 80% to 98% for plasma, and from 102% to from 112% to 97% to 109% for urine, respectively. The accuracy and precision of PQ and 5,6-PQ in plasma and urine were within the acceptance criteria. The samples should be kept in the freezer (−80 °C) and analyzed within 7 days due to the metabolite stability. This validated UHPLC-MS/MS method was beneficial for a pharmacokinetic study in subjects receiving PQ.

## 1. Introduction

Primaquine (PQ) is an 8-aminoquinoline (8-AQ) drug [1]. It is a currently used drug that has been approved for decades to treat and prevent the relapsing strains of human malaria (*P. vivax* and *P. ovale*) because of its tissue schizontocidal, gametocidal, and hypnozoidal activities. These activities prevent both malaria relapse and malaria transmission [2,3]. PQ is primarily metabolized to carboxyprimaquine (CPQ) by monoamine oxidase-A (MAO-A). CPQ has not been reported to exhibit antimalarial activity or hemolytic toxicity nowadays [4,5]. Even though the mechanism of PQ has not been elucidated, recent research reported that 5-hydroxy-primaquine (5-OH-PQ) produced hydrogen peroxide (H_2_O_2_) and reactive oxygen species, causing oxidative damage in the parasite, as well as hemolytic toxicity. PQ is metabolized by CYP2D6 to phenolic metabolites and changed to 5-OH-PQ, which is unstable in both oxygen and light. It is capable of redox cycling and terminates in the stable 5,6-orthoquinone primaquine (5,6-PQ). Therefore, 5,6-PQ was used as a representative marker for 5-OH-PQ and suitable for determining the amount of PQ active metabolites [3,6].

There have been many supported studies in which PQ activity was shown to be dependent on CYP2D6 activation. CYP2D6 was the key enzyme in PQ metabolism and generated metabolites that were involved in the liver-stage antimalarial activity [3,5,7,8]. Moreover, *CYP2D6* polymorphisms have influenced *P. vivax* malaria relapse and PQ pharmacokinetic parameters [5,9,10,11,12]. Therefore, it would be interesting to measure both PQ and 5,6-PQ concentrations in individuals with different *CYP2D6* genetic polymorphisms.

Several human pharmacokinetic studies reported the use of the high-performance liquid chromatography–tandem mass spectroscopy (HPLC-MS/MS) method for the measurement of PQ and CPQ levels [12,13,14,15]. One of them reported the method for measuring the 5,6-PQ level in both human plasma and urine [15]. Two studies reported about PQ and CPQ method validation [16,17]. One recent research reported the method validation for 5,6-PQ quantification in human erythrocytes [18]. This study aimed to develop and validate the measurements of both PQ and 5,6-PQ levels in human plasma and urine. The clinical application of the method was further used in a pharmacokinetic study of PQ.

## 2. Material and Methods

### 2.1. Chemicals

Primaquine diphosphate ((4-*N*-(6-methoxyquinolin-8-yl)pentane-1,4-diamine;phosphoric acid; MW = 455); (primaquine; MW = 259)) and 8-aminoquinoline (quinolin-8-amine; MW = 144), internal standard (IS), were from *Sigma-Aldrich* (St. Louis, MO, USA). 5,6-Orthoquinone primaquine dihydrobromide (8-((5-aminopentan-2-yl)quinoline-5,6-dione dihydrobromide; MW = 259.31) was from Toronto Research Chemicals (Canada). Primaquine phosphate was from the Government Pharmaceutical Organization (Thailand). HPLC-grade methanol, acetonitrile, and formic acid were from *Sigma-Aldrich* (St. Louis, MO, USA). Water was purified in a Milli-Q system (Millipore, Bedford, MA, USA).

### 2.2. Instrumentation and Chromatographic Conditions

The ultra-high-performance liquid chromatography–tandem mass spectrometry ((UHPLC-MS/MS) system (UltiMate^TM^ 3000 HPLC Systems and TSQ Quantum Access MAX, Thermo Fisher Scientific, MA, USA) comprised Rapid Separation (RS) pump, vacuum degasser, RS autosampler, RS column compartment, and triple-stage quadrupole mass spectrometer. The separation was performed using a Hypersil GOLD^TM^ aQ C_18_ column (100 × 2.1 mm, particle size 1.9 μm) with a C_18_ guard column ((4 mm × 3 mm) from Thermo Fisher (San Jose, CA, USA)). The column temperature was maintained at 25 °C. An isocratic mode of mobile phase A (0.1% of formic acid in methanol:water (40:60, *v*/*v*)) and mobile phase B (0.1% of formic acid in acetonitrile) flowed in a ratio of 80:20 at 0.4 mL/min. The injection volume was 1 µL.

Mass analysis with an electrospray ionization (ESI) system was performed with a spray voltage of 4.0 kV in a positive mode, a sheath gas nitrogen pressure of 40 (arbitrary units), an auxiliary nitrogen gas of 20 (arbitrary units), a vaporizer temperature of 350 °C, an ion transfer capillary temperature of 370 °C, and a skimmer offset of 15 V. For the characterization of PQ, 5,6-PQ, and 8-AQ, the collision gas was used at 1.5 mTor, and the collision energy was set to 25 eV for PQ (*m/z* = 260.26 > 187.82), to 33 eV for 5,6-PQ (*m/z* = 260.20 > 147.13), and to 24 eV for 8-AQ (*m/z* = 145.00 > 128.16). TSQ Tune software (version 2.6 SP1, Thermo Electron Corporation, Hemel Hempstead, UK) was used for the optimization of tuning parameters. LC Quan™ software (version 3.0, Thermo Electron Corporation, Hemel Hempstead, UK) was used for data acquisition and processing.

### 2.3. Standard Stock Solutions Preparation

Stock solutions of PQ, 5,6-PQ, and 8-AQ were prepared separately (1 mg/mL base in methanol) and protected from light at −80 °C. Working standard solutions were prepared from the primary stock at 2, 20, and 30 μg/mL. The PQ and 5,6-PQ standard curves (25–1500 ng/mL) were prepared by spiking blank plasma or urine and serially diluting to the desired concentration.

Internal standard (100 ng/mL) was added to all samples. Quality-control (QC) samples were prepared in blank plasma or urine at concentrations of low limit of quantitation (LLOQ), low quality control (LQC), middle quality control (MQC), and high quality control (HQC) (25, 50, 600, and 1245 ng/mL, respectively) and stored at −80 °C prior to use. 

### 2.4. Method Validation

The developed HPLC-MS/MS method was validated according to the FDA and EMA guidelines [19,20]. Selectivity and specificity were determined from six separate plasma or urine samples to demonstrate that blank and zero calibrators should be free of any interferences at the retention times of the analytes and the IS.

For linearity and sensitivity, the calibration curves of at least six concentrations (25–1500 ng/mL) were analyzed in triplicate and constructed by plotting the peak area ratio of the analyte and the IS against nominal concentration to demonstrate the linearity of the method. The coefficient of determination (r^2^) of the calibration curves for all interested analytes should be ≥0.99. The limit of detection (LOD) or the limit of quantification (LOQ) was defined as the concentration with a 3:1 or 10:1 signal-to-noise ratio, respectively. The LOD was calculated as means from five replicates. The LOQ was determined from the lowest concentration measurable with precision and accuracy ±20%.

Matrix effect was determined from the responses of PQ and 5,6-PQ QCs spiked into six different plasma or urine samples. The matrix effect was determined from [response of QCs spiked in post-extraction blank samples × 100]/[response of the QCs in pure solutions]. The matrix effect should be within ±20%. The %coefficient of variation (%CV) on matrix effects should not be greater than ±15%. 

The absolute recovery was determined from [response of extracted QCs in blank samples × 100]/[response QCs spiked in post-extraction blank samples]. The extent of the recovery of analytes and of the IS should be consistent and reproducible. The %CV on recovery should not be greater than ± 15%.

Accuracy and precision were determined from the intra-day and inter-day runs. The accuracy should be within ±15% of the nominal concentration at LQC, MQC, and HQC, but ±20% at LLOQ; and the precision (%CV) should be within ±15% at LQC, MQC, and HQC, but ±20% at LLOQ.

The stability of PQ and 5,6-PQ in the QC standard solutions and samples was only determined under specific conditions during analysis. Short-term stability was determined in an autosampler (15 °C for 24 h) and benchtop/room temperature (25 °C for 4 h). Long-term stability of PQ and 5,6-PQ were assessed in the QC standard solutions and samples that were kept at −80 °C to determine an optimal storage condition and total analysis time. The obtained results were compared with the nominal concentrations of the analyte, and it was considered stable if the observed concentrations were within ±15% of the nominal concentration of the nominal concentration.

### 2.5. Application of Method

#### 2.5.1. Subjects and Sample Collection

Samples from three healthy Thai subjects who were orally administered a single dose of PQ phosphate (30 mg base) were analyzed. Venous blood (3 mL) was collected from participants into a heparin tube at time point 0 (predose), 1, 2, 3, 4, 6, 8, 12, 18, and 24 h after drug administration. Plasma was separated by centrifuging at 5000× *g* for 5 min within 30 min of collection. Urine was also collected at time point 0 (predose), during 0–4, 4–8, 8–12, 12–18, and 18–24 h after drug administration. The separated plasma and urine aliquots (10 mL) were protected from light, kept on dry ice at −80 °C during transportation, and transferred for storage at −80 °C until LC-MS analysis [14,15,21,22]. 

#### 2.5.2. Sample Preparation

Plasma or urine (190 μL) was spiked with an IS 100 ng (10 μL) and briefly vortex mixed. They were added to 0.2 mL of acetonitrile, mixed, and centrifuged at 10,000× *g* for 5 min. The only supernatant of plasma was again added to 0.2 mL of acetonitrile, mixed, and centrifuged at 10,000× *g* for 5 min for protein precipitation a second time. Each plasma or urine supernatant was filtered and transferred to a vial with a glass insert before being injected into the LC-MS system. 

#### 2.5.3. Pharmacokinetic Parameters Analysis

The plasma pharmacokinetic parameters (maximum concentration [Cmax], time to Cmax [Tmax], the area under the curve [AUC], and the area under the curve with extrapolation to infinity [AUC_0-inf_] for PQ and 5,6-PQ were determined from the plasma concentration versus time data using the non-compartmental analysis and calculated using STATA software, version 15.1 (StataCorp LLC, College Station, TX, USA). The urine pharmacokinetic parameters (maximum concentration [Cmax], time to Cmax [Tmax], the amount of drug excreted (AE), and the cumulative amount of drug excreted (CAE)) were determined or calculated from the urine AE versus time data. Urine Tmax was the midpoint time of the urine collection interval. The AE was calculated by the summation of drug excreted (urine concentration (ng/mL) multiplied by urine volume (mL)) during the urine collection period. CAE was calculated by the accumulation amount of drug excreted after each collection interval. 

## 3. Results

### 3.1. Fragmentation Patterns and MS/MS Spectra of PQ and 5,6-PQ

**Figure 1:**
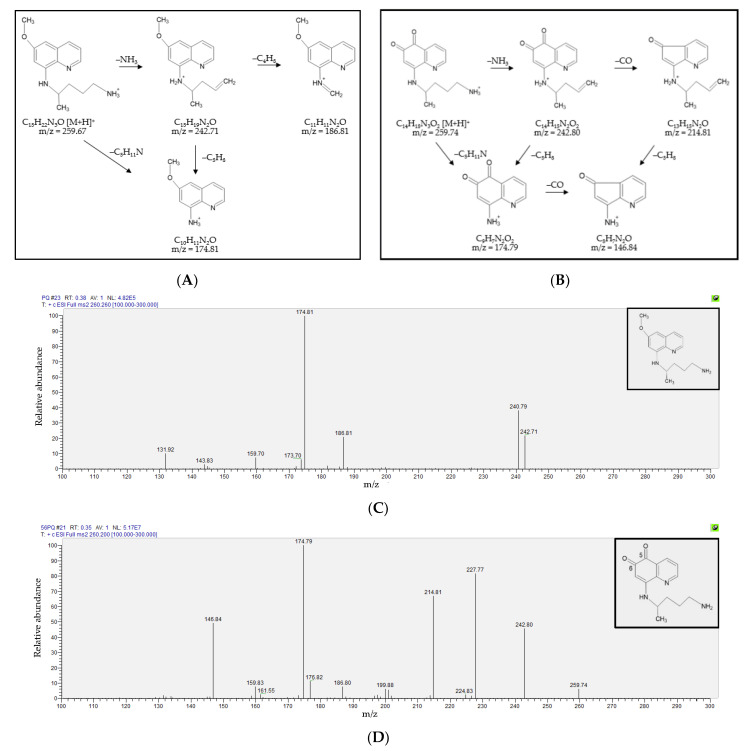
The fragmentation patterns and the MS/MS spectra of primaquine (PQ) and 5,6-orthoquinone primaquine (5,6-PQ). The fragmentation patterns of (**A**) PQ; and (**B**) 5,6-PQ. The MS/MS spectra of (**C**) PQ showed the key fragments at *m/z* 242.71 (−17; –NH_3_), 186.81 (−73; –C_4_H_11_N), and 174.81 (−85; –C_5_H_11_N); and (**D**) 5,6-PQ showed the key fragments at *m/z* 242.80 (−17; –NH_3_), 214.81 (−45; –CH_3_ON), 174.79 (−85; –C_5_H_11_N), and 146.84 (−113; –C_6_H_11_NO).

The fragmentation patterns and MS/MS spectra of PQ and 5,6-PQ were shown in Figure 1. The MS/MS spectra of PQ (*m/z* = 259.67) showed the key fragments at *m/z* 242.71 (−17; –NH_3_), 186.81 (−73; –C_4_ H_11_N), and 174.81 (−85; –C_5_H_11_N). The MS/MS spectra of 5,6-PQ (*m/z* = 259.74) showed the key fragments at *m/z* 242.80 (−17; –NH_3_), 214.81 (−45; –CH_3_ON), 174.79 (−85; –C_5_H_11_N), and 146.84 (−113; –C_6_H_11_NO).

### 3.2. Detection and Quantification of Primaquine, 5,6-Orthoquinone Primaquine and 8-Aminoquinoline

**Figure 2:**
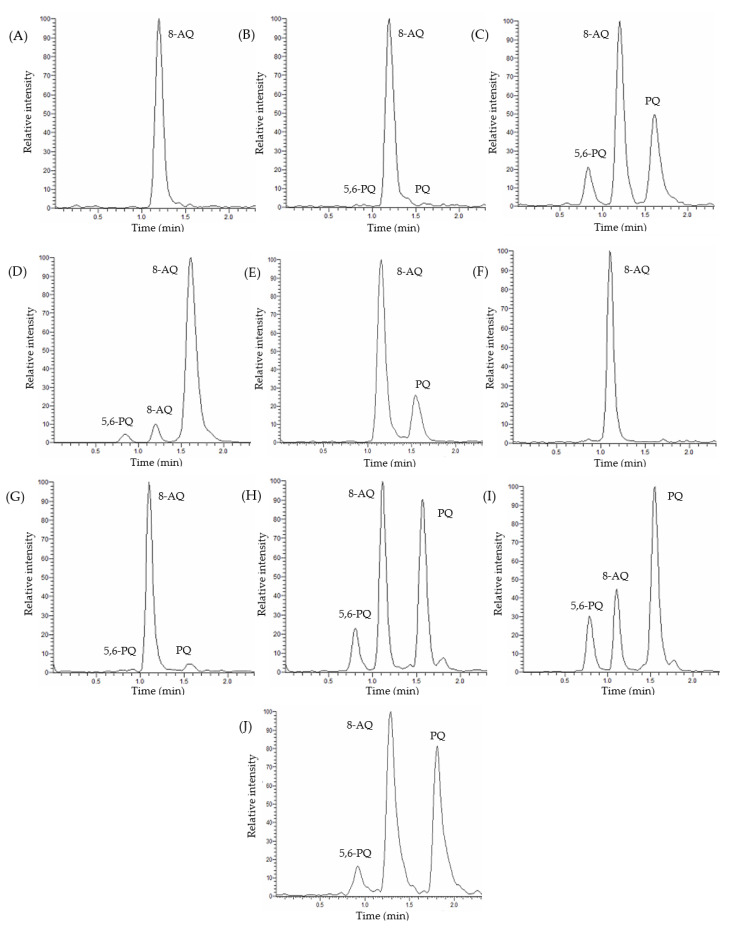
Chromatograms of 5,6-PQ, 8-aminoquinoline (8-AQ), and PQ in human plasma and urine. (**A**) Blank plasma with an internal standard (IS). (**B**) Spiked plasma at low limit of quantitation (LLOQ) for PQ and 5,6-PQ. (**C**) Spiked plasma at 600 ng/mL for PQ and 5,6-PQ. (**D**) Spiked plasma at 1500 ng/mL for PQ and 5,6-PQ. (**E**) A clinical trial plasma sample. (**F**) Blank urine with an IS. (**G**) Spiked urine at LLOQ for PQ and 5,6-PQ. (**H**) Spiked urine at 600 ng/mL for PQ and 5,6-PQ. (**I**) Spiked urine at 1500 ng/mL for PQ and 5,6-PQ. (**J**) A clinical trial urine sample.

The retention times of 5,6-PQ, 8-AQ, and PQ in plasma and urine were 0.8, 1.2, and 1.6 min, respectively. Representative chromatograms for each analyte in human plasma and urine are shown in Figure 2.

### 3.3. Method Validation

#### 3.3.1. Selectivity and Specificity 

No significant peak interfered with the quantification of PQ and 5,6-PQ in the chromatograms of blank human plasma and urine.

#### 3.3.2. Linearity and Sensitivity

The coefficient of determination (r^2^) values were ≥0.99 for all calibration curves. The LODs of PQ and 5,6-PQ in plasma were 10.74 ± 4.32 and 2.99 ± 1.82 ng/mL, respectively. The LODs of PQ and 5,6-PQ in urine were 8.77 ± 3.74 and 1.05 ± 0.59 ng/mL, respectively. The LOQs of PQ and 5,6-PQ in human plasma and urine were 25 ng/mL. Chromatograms of blank samples with IS, spiked samples at LLOQ, MQC, upper limit of quantitation (ULOQ), and clinical trial samples are shown in Figure 2. The selected reaction monitoring (SRM) chromatograms of PQ and 5,6-PQ at LLOQ in plasma and urine are shown in Supplementary Figures S1 and S2.

#### 3.3.3. Matrix Factor and Recovery 

The matrix effect of all analytes was between 80% and 120% according to the acceptance criteria. The average matrix effects for PQ and 5,6-PQ in human plasma were range from 100% to 116% and from 87% to 104%, respectively. The average matrix effects for PQ and 5,6-PQ in human urine ranged from 87% to 89% and from 86% to 87%, respectively. The results indicated that there were no matrix effects as presented in Table 1.

The recovery of all analytes was consistent and reproducible according to the acceptance criteria. The average recoveries for PQ and 5,6-PQ in human plasma ranged from 78% to 95% and from 80% to 98%, respectively. The average recoveries for PQ and 5,6-PQ in human urine ranged from 102% to 112% and from 97% to 109%, respectively. This extraction method was suitable for sample preparation as shown in Table 1.

#### 3.3.4. Accuracy and Precision 

The accuracy values of PQ and 5,6-PQ in human plasma and urine were within the acceptance criteria of ± 20% of LLOQ and ± 15% of the QCs. The precision (%CV) values did not exceed 20% for the LLOQ and 15% for each QC as shown in Table 2.

#### 3.3.5. Stability 

The concentration of PQ and 5,6-PQ in QC standard solutions and samples after the short-term stability test changed less than ±15% within the specified time, indicating that all analytes were stable during and while waiting for analysis. Moreover, in the long-term stability test, the concentration of standard solutions changed less than ± 15% within 7 days. The concentration of PQ and 5,6-PQ in QC standard solutions and samples kept at −80 °C also changed less than ±15% within 7 days, indicating that all analytes were stable during storage in the freezer and that the samples should be used for analysis within 7 days.

### 3.4. Application of Method

**Figure 3:**
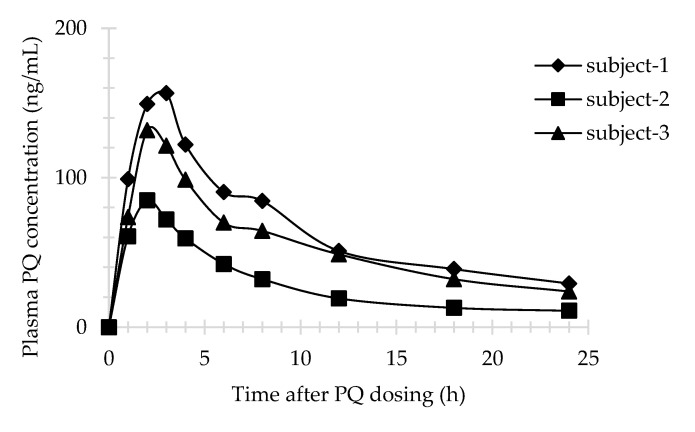
PQ in human plasma (*n* = 3); Subject-1, 44 years old (diamond line); Subject-2, 55 years old (square line); Subject-3, 49 years old (triangle line).

**Figure 4:**
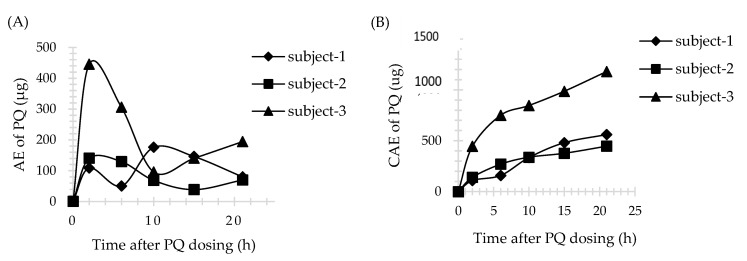
PQ in human urine (*n* = 3). (**A**) The amount of drug excreted (AE) versus time profile; (**B**) The cumulative amount of drug excreted (CAE) versus time profile. Time was the midpoint time of the urine collection interval.

**Figure 5:**
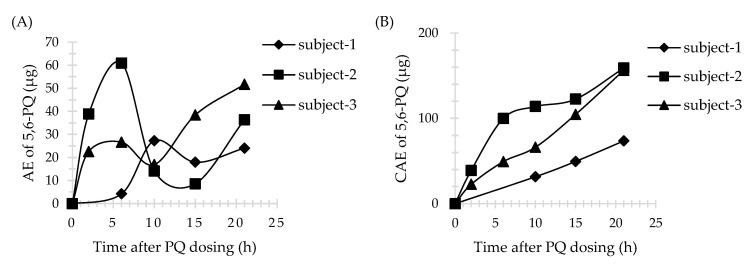
5,6-PQ in human urine (*n* = 3). (**A**) AE versus time profile. (**B**) CAE versus time profile. Time was the midpoint time of the urine collection interval.

Three male subjects received a single dose of 30 mg PQ. The plasma profile of PQ is shown in Figure 3. All plasma concentrations were above LLOQ and within the calibration curve range for the assay. However, 5,6-PQ was undetectable in plasma samples. The urine profiles of PQ and 5,6-PQ are shown in Figure 4 and Figure 5, respectively. The pharmacokinetic parameters in human plasma and urine are shown in Table 3 and Table 4, respectively.

## 4. Discussion

This research reported the development and validation of a simplified, rapid, and reliable UHPLC-MS/MS method for determination of PQ and 5,6-PQ in human plasma and urine. This was the first validation report of the 5,6-PQ level measurements in these samples. This method was adapted from Page-Sharp et al. [17] as follows: the use of the UHPLC column reduced the separation time of each analyte to less than 2 min, while several LC-MS studies all reported separation times and retention times of PQ more than 10 min [14,16,17] and 3 min [16,17], respectively. Acetonitrile addition as a mobile phase increased the flow rate, and mobile phase ratio adjustment among methanol, water, and acetonitrile allowed the measurement of PQ, 5,6-PQ, and 8-AQ faster, simultaneously, and also greatly shortened the analysis time. Moreover, the use of low injection volume (1 μL) to the LC-MS system was useful when the sample volume was small compared to the use of a larger volume (10 μL) from the other studies [14,17].

The samples were extracted using only acetonitrile. The %recovery analysis of all analytes was within optimal range and similar to the previous study [17,18]. This simple extraction method could reduce the sample preparation time prior to analysis. The matrix effect analysis of PQ was in the range of 80–120% and compared favorably with the other previous study [17]. The LOQ values of PQ and 5,6-PQ in plasma and urine were 25 ng/mL. This study has a certain level of sensitivity compared to the study using the quadrupole mass spectrometer [17]. The LOQ value of PQ from Avula et al. showed better characteristics compared to this study. The use of a quadrupole-time-of-flight (*Q-TOF*) tandem *mass spectrometer* [16] or a single time-of-flight mass spectrometer might increase the sensitivity. The lower LOQ value of 5,6-PQ in erythrocytes from Khan et al. showed better sensitivity compared to that in the plasma in this study. However, the matrices that were used to compare among these methods were different (erythrocyte versus plasma, and urine samples). Red blood cell extraction could be more complex and time-consuming compared to this extraction method [18]. The recovery, matrix effect, accuracy, and precision values of PQ and 5,6-PQ from this method were similar to those in the other studies [16,17,18]. Due to the stability of the PQ and 5,6-PQ, the concentration was changed to less than ±15% during analysis. The sample should be stored in the freezer (−80 °C) and accelerated for analysis within 7 days. Quantification in each batch of analysis should be concerned with the number of subjects, transportation time, sample preparation time, and all analytical times. The method validation parameters compared with the other studies are shown in Table 5.

This method was further used for the determination of both PQ and 5,6-PQ in human plasma and urine in a pharmacokinetic study. It is particularly suitable for the quantification of PQ and 5,6-PQ in urine samples. The higher LOQ value from this study might cause 5,6-PQ to be undetectable in real plasma samples. However, the use of a high-sensitivity mass spectrophotometer such as a triple quadrupole or Q-TOF (the LOQ values have not been reported) could not detect or quantify this metabolite in plasma [14,15]. It is possible that 5,6-PQ might not be suitable for plasma quantification. The pharmacokinetic study of PQ in mice showed that 5,6-PQ concentration was higher in the liver than in plasma. It was rapidly excreted from the liver and blood circulation [3], consistent with the specific action of this metabolite found mainly in the liver.

Sample data from healthy volunteers who had received a single dose of 30 mg PQ (0.35–0.46 mg/kg/dose) were used to generate plasma concentration versus time profile and urine AE or CAE versus time profile and to calculate pharmacokinetic parameters. There were only two previous pharmacokinetic studies of healthy volunteers who received a single dose of 30 mg PQ. The pharmacokinetic parameters of PQ in plasma from Mihaly et al., Cmax, Tmax, and AUC_0-inf_, were in the range of 124–249 ng/mL, 2.4–2.5 h, and 1166–2947 ng/mL*h, respectively. The Cmax and Tmax in urine were in the range of 500–800 ng/mL and 2 h for PQ, and 25.2–762 ng/mL and 2–18 h for 5,6-PQ, respectively [23]. Moreover, the Cmax, Tmax, and AUC_0-inf_ of PQ in plasma from Spring et al., were in the range of 79–129 ng/mL, 2–4 h, and 1000–1400 ng/mL*h, respectively [15]. The pharmacokinetic result in this study showed interpersonal variation. Subject no.2 had different pharmacokinetic parameters of PQ in plasma, while subject no.3 and no.1 had different pharmacokinetic parameters of PQ and 5,6-PQ in urine, respectively, compared with the two others. The genetics involved in PQ pharmacokinetics might be the reason for this event. Unfortunately, the genetic profile of these three subjects was not evaluated.

PQ activity was shown to be dependent on CYP2D6 activation. 5-OH-PQ, the active metabolite involved in the liver-stage antimalarial activity as well as hemolytic toxicity, was generated by CYP2D6 and subsequently changed into 5,6-PQ, the stable form of 5-OH-PQ [3,5,6,7,8]. Previous studies have reported determining the relationship between CYP2D6 polymorphisms and PQ, CPQ concentrations. However, CPQ is not mainly metabolized by CYP2D6. It is an inactive metabolite from MAO-A. Therefore, the method of measuring the 5,6-PQ level would be interesting and useful for further PQ pharmacokinetic studies in subjects with differences in CYP2D6 enzyme activity. 

## 5. Conclusions

A simplified, rapid, and reliable UHPLC-MS/MS method for determination of PQ and 5,6-PQ in human plasma and urine has been developed and validated. This was the first validation report of the 5,6-PQ level measurements in human plasma and urine. The method of 5,6-PQ measurement was beneficial for applying in further pharmacokinetic studies of subjects receiving PQ.

## Figures and Tables

**Table 1:** Matrix effect (*n* = 6) and recovery (*n* = 9) analysis of PQ and 5,6-PQ in human plasma and urine.

Analytes	Human Plasma		Human Urine	
	Matrix Effect (%) (mean ± SD/CV)	Recovery (%) (mean ± SD/CV)	Matrix Effect (%) (mean ± SD/CV)	Recovery (%) (mean ± SD/CV)
PQ				
LQC	100 ± 16/6.38	77.8 ± 8.91/11.45	87 ± 5/1.94	108.39 ± 9.92/9.15
MQC	-	95.30 ± 13.95/14.64	-	111.91 ± 3.45/3.08
HQC	116 ± 5/2.04	91.59 ± 3.60/3.93	89 ± 3/1.65	102.24 ± 9.51/9.30
5,6-PQ				
LQC	104 ± 12/7.11	97.93 ± 12.62/12.88	86 ± 5/2.33	97.49 ± 8.17/8.38
MQC	-	84.58 ± 12.45/14.72	-	108.00 ± 3.24/3.00
HQC	87 ± 2/4.88	80.47 ± 5.72/7.11	87 ± 3/2.38	108.51 ± 2.70/2.49

LQC: low quality control, MQC: middle quality control, HQC: high quality control, SD: standard deviation, CV: coefficient of variation (CV = [SD × 100]/mean). -: Not tested.

**Table 2:** Accuracy and precision analysis of PQ and 5,6-PQ in human plasma and urine.

Analytes	Human Plasma		Human Urine	
	Intra-Day (*n* = 5) Accuracy/Precision (%, mean ± SD/%, CV)	Inter-Day (*n* = 15) Accuracy/Precision (%, mean ± SD/%, CV)	Intra-Day (*n* = 5) Accuracy/Precision (%, mean ± SD/%, CV)	Inter-Day (*n* = 15) Accuracy/Precision (%, mean ± SD/%, CV)
PQ				
LLOQ	98.32 ± 2.18/2.22	99.17 ± 7.65/3.04	108.66 ± 1.12/1.03	102.62 ± 7.4/3.22
LQC	112.08 ± 1.54/1.37	103.98 ± 5.95/3.76	97.19 ± 6.31/6.49	99.46 ± 3.27/4.70
MQC	92.05 ± 4.63/5.03	94.45 ± 0.72/5.90	105.02 ± 7.8/7.43	98.14 ± 6.06/5.85
HQC	100.46 ± 1.84/1.83	100.52 ± 2.11/3.10	107.58 ± 2.93/2.72	95.46 ± 10.89/3.44
5,6-PQ				
LLOQ	94.35 ± 1.72/1.82	100.47 ± 11.82/2.27	114.18 ± 1.68/1.47	109.52 ± 7.74/4.13
LQC	99.56 ± 3.98/4.00	97.98 ± 6.90/6.55	112.82 ± 4.24/3.76	111.42 ± 1.7/7.76
MQC	90.10 ± 10.98/12.19	89.19 ± 1.01/7.83	94.82 ± 4.78/5.04	92.41 ± 2.37/9.13
HQC	93.66 ± 6.34/6.77	93.24 ± 4.29/6.95	94.53 ± 5.3/5.61	94.18 ± 2.64/4.51

**Table 3:** Pharmacokinetic parameters of PQ in human plasma (*n* = 3).

Subject ID	BW (kg)	BMI (kg/m^2^)	Cmax (ng/mL)	Tmax (h)	AUC (ng/mL*h)	AUC_0-inf_ (ng/mL*h)
1	86	28.08	156.62	3	1597.57	1613.65
2	67.2	26.25	84.84	2	693.67	709.54
3	65	25.71	131.73	2	1315.37	1326.87

ID: identification, BW: body weight, BMI: body mass index (BMI = *weight* (kg)/[*height* (m)]*^2^*), Cmax: maximum concentration, Tmax: time to Cmax, AUC: area under the curve, AUC_0-inf_: area under the curve with extrapolation to infinity.

**Table 4:** Pharmacokinetic parameters of PQ and 5,6-PQ in human urine (*n* = 3).

Subject ID		PQ			5,6-PQ	
	AEmax (μg)	Tmax (h) ^a^	CAE (μg)	AEmax (μg)	Tmax (h) ^a^	CAE (μg)
1	86	28.08	156.62	3	1597.57	1613.65
2	67.2	26.25	84.84	2	693.67	709.54
3	65	25.71	131.73	2	1315.37	1326.87

^a^ Tmax was the midpoint time of the urine collection interval.

**Table 5:** Comparison of method validation parameters between this study and the others.

Parameters	Analytes	Avula et al., 2011 [16]	Page-Sharp et al., 2012 [17]	Khan et al., 2021 [18]	This Study	
**LOD**	PQ 5,6-PQ	2 ng/mL N/D	1 μg/mL N/D	N/D 1.62 ± 0.23 ng/mL	10.74 ± 4.32 ng/mL 2.99 ± 1.82 ng/mL	8.77 ± 3.74 ng/mL 1.05 ± 0.59 ng/mL
**LOQ**	PQ 5,6-PQ	5 ng/mL N/D	2 μg/mL N/D	N/D 4.95 ± 0.61 ng/mL	25 ng/mL	
**Recovery**	PQ 5,6-PQ	87–88% N/D	79–117% N/D	N/D 84–98%	78–95% 80–98%	102–112% 97–109%
**Matrix effect**	PQ 5,6-PQ	N/D	97–118% N/D	N/D	100–116% 87–104%	87–89% 86–87%
**Accuracy**	PQ 5,6-PQ	101–112% N/D	94–124% N/D	N/D 96–103.2%	92–112% 90–100%	95–109% 92–114%
**%RSD**	PQ 5,6-PQ	0.2–9.1% N/D	4.8–9.7% N/D	N/D 5.49%	1.37–3.9% 1.82–12.19%	1.03–7.43% 1.47–7.76%
**Stability**	PQ 5,6-PQ	N/D	N/D	N/D 13 days	7 days	
**Matrix**		Mice plasma	Human plasma	Human erythrocyte	Human plasma	Human urine

LOD: limit of detection, LOQ: limit of quantification, RSD: relative standard deviation (%RSD = coefficient of variation), N/D: no data.

## Data Availability

Data is contained within the article or Supplementary Materials.

## Supplementary Materials

The following are available online, Figure S1. The SRM chromatograms of PQ and 5,6-PQ at LLOQ in plasma. Figure S2. The SRM chromatograms of PQ and 5,6-PQ at LLOQ in urine.

## Sample Availability

Samples of compounds are not available from the authors.
