# Supplementary material for: Simplified and Rapid Determination of Primaquine and 5,6-Orthoquinone Primaquine by UHPLC-MS/MS: Its Application to a Pharmacokinetic Study
Source: Molecules. 2021 Jul 19;26(14):4357. doi: 10.3390/molecules26144357 (PMC8304466; doi:10.3390/molecules26144357)

Supplementary Materials

Simplified and rapid determination of primaquine and 5,6-*ortho*-quinone-primaquine by UHPLC-MS/MS : its application to a pharmacokinetic study

Waritda Pookmanee [1,2], Siriwan Thongthip [3], Jeeranut Tankanitlert [4], Mathirut Mungthin [4], Chonlaphat Sukasem [5,6,7] and Supeecha Wittayalertpanya [2,8,*]

[1]Interdisciplinary Program in Pharmacology, Graduate School, Chulalongkorn University, Bangkok 10330, Thailand;
[2]Clinical Pharmacokinetics and Pharmacogenomics Research Unit, Faculty of Medicine, Chulalongkorn University, Bangkok 10330, Thailand
[3]Maha Chakri Sirindhorn Clinical Research Center Under the Royal Patronage, Faculty of Medicine, Chulalongkorn University, Bangkok 10330, Thailand;
[4]Department of Pharmacology, Phramongkutklao College of Medicine, Bangkok 10400, Thailand;
[5]Division of Pharmacogenomics and Personalized Medicine, Department of Pathology, Faculty of Medicine Ramathibodi Hospital, Mahidol University, Bangkok 10400, Thailand;
[6]Laboratory for Pharmacogenomics, Somdech Phra Debaratana Medical Center (SDMC), Ramathibodi Hospital, Bangkok 10400, Thailand
[7]Pharmacogenomics and Precision Medicine, The Preventive Genomics & Family Check-up Services Center, Bumrungrad International Hospital, Bangkok 10110, Thailand
[8]Department of Pharmacology, Faculty of Medicine, Chulalongkorn University, Bangkok 10330, Thailand

[*]Correspondence: supeechas@hotmail.com; Tel.: +668-1421-9164

Figure S1. The SRM chromatograms at LLOQ in plasma; (A) PQ; and (B) 5,6-PQ.

(A)

(B)

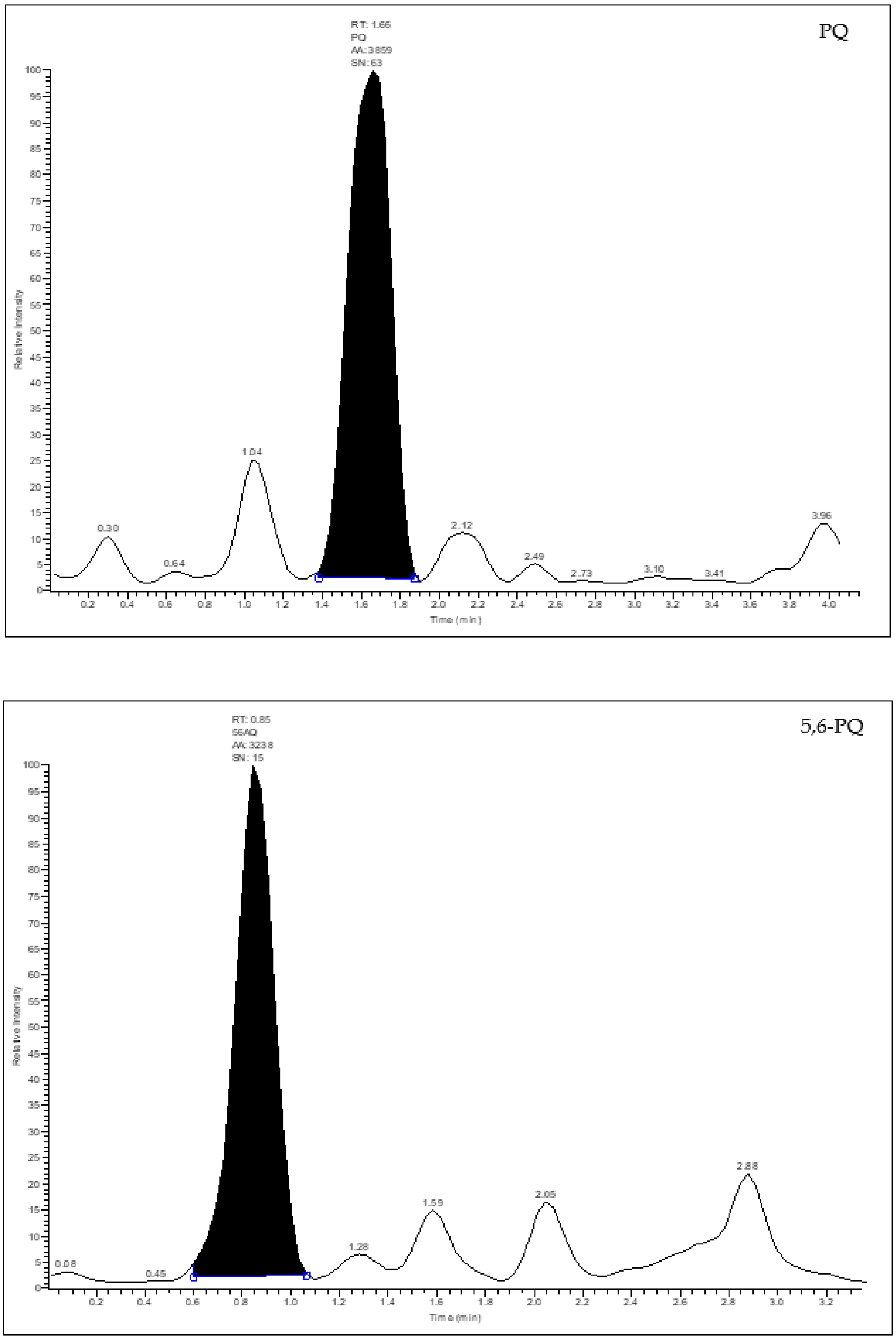

Figure S2. The SRM chromatograms at LLOQ in urine; (A) PQ; and (B) 5,6-PQ.

(A)

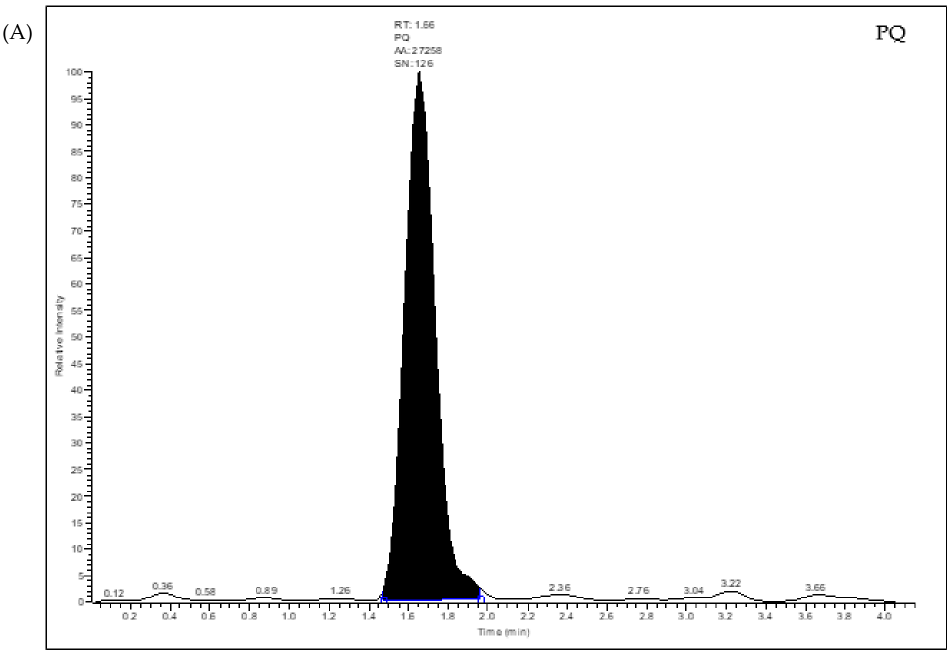

(B)

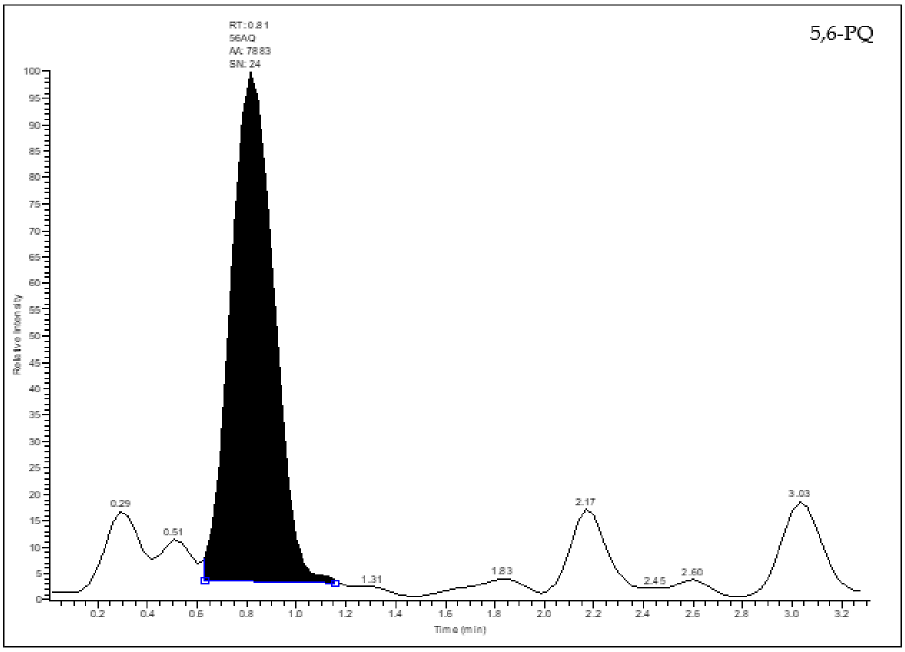

Supplement: Supplementary file 1 [file molecules-26-04357-s001.zip › molecules-1268028-supplementary.pdf]
